# Subtemporal transtentorial approach for resection of a pontomesencephalic cavernous malformation

**DOI:** 10.3171/2019.10.FocusVid.19365

**Published:** 2019-10-01

**Authors:** Georgios A. Zenonos, Samir Sur, Maximiliano Nuñez, David T. Fernandes-Cabral, Jacques J. Morcos

**Affiliations:** 1 Department of Neurological Surgery, University of Miami, Miami, Florida, and; 2 Department of Neurological Surgery, University of Pittsburgh Medical Center, Pittsburgh, Pennsylvania

**Keywords:** subtemporal transtentorial approach, cavernous malformation, safe entry zones, brainstem anatomy, video

## Abstract

In this 3D video we review the case of a pontomesencephalic cavernous malformation in a 27-year-old woman who presented with hemiparesis and diplopia. The cavernous malformation was completely resected through a subtemporal transtentorial approach and an epitrigeminal brainstem entry zone, with a significant improvement in the patient’s hemiparesis. The relevant anatomy is reviewed in detail through multiple anatomical brainstem dissection specimens, as well as high-definition fiber tractography images. The rationale for the approach is analyzed relative to other possible options, and a number of technical pearls are provided.

The video can be found here: https://youtu.be/8EoIWL7XqAc.

## Transcript

### 0:20 Introduction—Presentation

In this video we will present the subtemporal transtentorial approach for resection of a pontomesencephalic cavernous malformation. The case refers to a 27-year-old female who had a fall 10 days prior to presentation, followed by progressive weakness and numbness, as well as diplopia and difficulty speaking.

On physical examination she was wide awake, alert and oriented, but dysarthric, with left-sided central facial weakness and left-sided facial hemianesthesia. On examination of her extraocular movements, she could only abduct her right eye, consistent with a one-and-a-half medial longitudinal fasciculus syndrome, as well as dysfunction of the rostral interstitial nucleus of the MLF. In addition, she had left-sided hemiparesis, with the arm affected more than the leg, and left body hemianesthesia.

### 1:09 Imaging

A T2 MRI showed a hemorrhagic lesion spanning the upper pons and lower midbrain, and which came closer to the surface right above the trigeminal entry zone. The lesion appeared mostly hemorrhagic, but with some solid components at its inferomedial margin, which were more consistent with the popcorn appearance of a cavernous malformation. No other vascular lesion was seen on the contrasted images, and interestingly, we also did not appreciate a developmental venous anomaly.

### 1:37 Anatomy review

It is useful to review some of the relevant brainstem anatomy of the upper pons, where most of the solid components of the lesion are located. The crus cerebri projections traversing the pontomesencephalic fissure consist mainly of corticospinal fibers, which are straddled by frontopontine and temporopontine fibers medially and laterally, respectively. These frontopontine and temporopontine fibers are almost as prominent as the corticospinal tracts in the upper pons, and together they occupy the majority of the basis pontis. Notably, the corticobulbar fibers, including those heading towards the contralateral abducens nucleus, course with the medial corticospinal tracts. In the pontine tegmentum, we find the medial longitudinal fasciculus, the mesencephalic nucleus of five, the tectospinal tracts, and the superior cerebellar peduncle. In between, and from lateral to medial, we find the lateral lemniscus carrying the auditory projections, the spinothalamic tracts carrying general sensory fibers, as well as the medial lemniscus carrying the projections from the dorsal columns. The rostral interstitial nucleus of the MLF, also known as the vertical gaze center, is located along the medial longitudinal fasciculus but slightly higher, at the level of the tectum. It is easier now to understand how involvement of most of these structures by the hematoma explains our patient’s symptoms.

### 2:57 Choice of approach

Again our hematoma occupies the lower pons, extending into the lower midbrain, while it comes closer to the surface just above the trigeminal entry zone. One option is to perform a retrosigmoid craniotomy and enter the hematoma through a lateral transpeduncular entry zone. However, this approach would be ill-suited for approaching the solid components of the lesion, which were seen at its inferomedial margin. Another option, which was the one we favored, was to come through a subtemporal transtentorial approach and enter the hematoma through an epitrigeminal entry zone. This approach would provide optimal access to all components of the lesion, and the best visualization of the inferomedial solid components.

### 3:35 Approach-related anatomy review

It is useful to remember that the crus cerebri projections rotate outwards as they descend in the pons. So when seen from a subtemporal transtentorial approach, the epitrigeminal entry zone courses behind the corticospinal tracts, only possibly disrupting some of the temporopontine fibers, while it is directly anterior to the spinal lemniscus and the spinothalamic tracts.

### 3:55 Positioning

The patient is positioned supine, with a bump under her right shoulder. Her head was turned so that her temple was parallel to the floor and tilted down. A linear incision was marked to accommodate a craniotomy which was two-thirds anterior and one-third posterior to the external auditory canal. Somatosensory, motor, and auditory brainstem evoked potentials were monitored, as well as EMGs for cranial nerves III, IV, and V. A lumbar drain was also placed preoperatively to achieve brain relaxation.

### 4:20 Approach

After incision, the temporalis is mobilized anteriorly, and the craniotomy is made flush with the middle fossa floor. Once the dura is opened, telfa patties are placed to protect the temporal lobe. One should be extremely careful, as the vein of Labbé consists of two or more individual veins that can drain directly in the tentorium. Here there were three, and the most medial one had to be sacrificed as it was right in the middle of our exposure. Coming all the way down to the tentorial incisura, the ambient cistern is opened, releasing CSF. The posterior cerebral artery as well as the trochlear nerve come into view. The oculomotor nerve is also dissected. A self-retaining retractor is placed to increase the exposure, but then we noticed that one of the veins of Labbé was being stretched. This was covered with wet Gelfoam to prevent desiccation, and the retractor was repositioned. We then started cutting the tentorium from lateral to medial, taking care to avoid injury to the trochlear nerve at the incisura. The point of entry of the nerve to the tentorium was identified, but we also saw a variant tentorial artery of Davidoff and Schechter. It is important to avoid avulsing this artery as this can create a side hole in the parent artery that may be difficult to control without injury to the parent vessel. To further increase the exposure, we then make a second tentorial cut, roughly parallel to the superior petrosal sinus. The tentorium can then be reflected and secured with an aneurysm clip.

### 6:07 Cavernoma resection

After confirming our location and trajectory with image guidance, the surface of the brainstem is stimulated to ensure absence of aberrant corticospinal fibers displaced by the hematoma. A small opening in the epitrigeminal entry zone is then made sharply, following the estimated trajectory of the transverse pontine fibers. This opening is developed with a Rhoton No. 6 bluntly releasing the hematoma. Some of the liquefied components of the hematoma are aspirated, whereas some more formed thrombi are evacuated in a piecemeal fashion. We then start to make our way to the inferomedial margin, where we had expected the more solid components of the cavernoma. Indeed, we appreciate the change in texture and start dissecting the mulberry-like lesion off the surrounding brainstem. Both sharp and blunt dissecting tools can be used to develop this plane. Small feeders to the cavernoma are coagulated carefully and then divided. These inferomedial solid components are then removed en bloc. Careful inspection of the cavity reveals a vascular structure in the depth. As discussed in the beginning, we did not appreciate a developmental venous anomaly on preoperative imaging; nevertheless, in cavernous malformations which present with large bleeds occasionally they can be thrombosed. As these components of the DVA can be a nidus for recurrence, this vascular structure is coagulated and resected. The final inspection of the walls reveals no concern for residual cavernoma.

### 7:33 Closure

The vein of Labbé was preserved throughout the case. The dura was closed in a watertight fashion followed by replacement of the bone flap.

### 7:58 Postoperative imaging and clinical course

Postoperative imaging confirmed a complete resection, and the patient had an immediate improvement in her hemiparesis, regaining at least antigravity strength.

**Correspondence** Georgios A. Zenonos, University of Miami, Miami, FL. george.zenonos@gmail.com.

## Disclosures

The authors report no conflict of interest concerning the materials or methods used in this study or the findings specified in this publication.
